# Patterns and Potential Relationships of Organizational Silence Types Among Clinical Nurses: A Qualitative Meta‐Synthesis and Systematic Review

**DOI:** 10.1002/nop2.71004

**Published:** 2026-11-03

**Authors:** Honghu Chen, Xuan Cheng, Jiayu Liao, Jiayu Hou, Jiayi Liu, Weihong Wang

**Affiliations:** ^1^ School of Nursing Hunan Normal University Changsha Hunan China

## Abstract

**Aims:**

To synthesize qualitative evidence on organizational silence among clinical nurses and examine relationships among motives, earlier organizational responses, and subsequent communication choices.

**Design:**

A qualitative systematic review and meta‐synthesis.

**Review Methods:**

One reviewer screened titles, abstracts, and full texts. A second reviewer verified extracted data. Two reviewers independently coded findings and appraised quality using the Joanna Briggs Institute qualitative checklist. Thomas and Harden's thematic synthesis was applied, followed by interpretation using social information processing theory.

**Data Sources:**

Scopus, Embase, PubMed, PsycINFO, CINAHL, China National Knowledge Infrastructure (CNKI), Wanfang Data, and the Chinese Scientific Journals Database were searched from inception to 6 October 2026. Initial searches were completed on 25 April 2026. Grey literature was not systematically searched.

**Results:**

Nineteen reports yielded four analytical themes encompassing 12 subthemes: (1) Motives distinguished forms of organizational silence; (2) Earlier responses informed later expression and participation; (3) Communication route and timing shaped the distinction between expression and withholding; and (4) Organizational conditions constrained expression across motives. Nurses' accounts linked withholding to anticipated adverse consequences, perceived futility, and efforts to preserve working relationships. The synthesis distinguished delayed or indirect communication from continued withholding.

**Conclusion:**

Established silence types describe motives rather than fixed categories of nurses. Shared contexts may inform different motives, but the synthesis does not establish transitions or causal pathways.

**Implications for Nursing Practice:**

Clear escalation pathways, constructive responses from leaders, timely feedback, and a psychologically safe work environment may help nurses offer suggestions and voice work‐related concerns. These pathways should facilitate prompt escalation of urgent patient safety concerns.

**Impact:**

The review addresses the interpretation of nurses' silence and communication choices. Findings clarify motivational boundaries and alternative expression, informing clinical supervision, debriefing, and speaking‐up systems for nurses and clinical leaders.

**Reporting Method:**

Preferred Reporting Items for Systematic Reviews and Meta‐Analyses 2020, supplemented by Enhancing Transparency in Reporting the Synthesis of Qualitative Research.

**Patient or Public Contribution:**

No patients or members of the public were involved in this systematic review and qualitative meta‐synthesis because the study was based exclusively on previously published research.

**Trial Registration:**

International Prospective Register of Systematic Reviews: CRD420261379111

## 1 Background

Organizational silence, a concept initially introduced by Morrison and Milliken (2000), refers to the phenomenon in which employees deliberately withhold their genuine opinions, concerns, or suggestions regarding work‐related issues due to a variety of individual and contextual reasons. In the nursing context, organizational silence is defined as the behaviour in which nurses, despite being equipped with professional knowledge and clinical experience that enable them to offer constructive feedback on nursing practice, patient safety, and ward management, nevertheless choose to remain silent owing to individual, organizational, or environmental constraints (Yang and Yang 2017). Within clinical settings, such silence not only undermines nurses' work engagement, professional identity, and organizational commitment, but also impedes effective team communication and collaboration, hampers continuous quality improvement and organizational innovation, and may, in severe cases, escalate patient safety risks (Zhao 2019; Long et al. 2024). Understanding organizational silence among nurses therefore requires examining not only their behaviour but also the underlying motives and the contexts in which silence occurs.

A previous qualitative meta‐synthesis identified defensive, acquiescent, disregardful, and prosocial silence among nurses and described their characteristics and consequences (Zou et al. 2025). A subsequent systematic review and meta‐analysis estimated the level of organizational silence among clinical nurses using quantitative studies (Wen et al. 2025). The present review examines the motives and contexts associated with organizational silence among clinical nurses and explores potential connections among established silence types. It also synthesizes accounts of how earlier organizational responses relate to later expression and participation, and distinguishes continued withholding from changes in the timing or route of communication.

Social information processing theory was used to interpret how nurses' interactions with leaders and colleagues may inform these patterns (Salancik and Pfeffer 1978). It was applied after inductive theme development and did not determine the initial codes or silence categories. The connections proposed here arise from comparisons across studies; they are not observed transitions between silence types within individual nurses.

## 2 Methods

### 2.1 Study Design and Approach

This study was designed as a qualitative systematic review and meta‐synthesis. Thomas and Harden's thematic synthesis (Thomas and Harden 2008) was used to integrate qualitative evidence on clinical nurses' organizational silence while preserving the distinction between participant accounts, primary‐study authors' interpretations, and review‐team analytical themes. Reporting was guided by the Preferred Reporting Items for Systematic Reviews and Meta‐Analyses 2020 (Page et al. 2021) and the Enhancing Transparency in Reporting the Synthesis of Qualitative Research statement (Tong et al. 2012). The reporting checklist, including manuscript locations and completion notes, is provided in Table S1.

### 2.2 Protocol Registration

This study was prospectively registered with the International Prospective Register of Systematic Reviews (PROSPERO) under registration number CRD420261379111 (available at the PROSPERO website). The detailed protocol can be accessed via the PROSPERO online platform.

### 2.3 Search Strategy

A comprehensive literature search was initially completed on 25 April 2026 in seven electronic databases: Scopus, Embase, PubMed, PsycINFO, CNKI, Wanfang Data, and VIP. In response to reviewer comments, the English‐language search strategies were revised and expanded, and CINAHL was added as an additional information source. The revised searches were completed on 6 October 2026, with the final search covering all eight databases from inception to that date.

English and Chinese search terms were adapted to each database's indexing and field structure. Compared with the initial strategies, the revised English‐language strategies incorporated broader nursing terms, alternative spellings and expressions related to organizational silence and speaking up, and a wider range of qualitative‐design terms. Nursing terms were combined with either organizational‐silence terms or speaking‐up terms paired with qualitative‐design terms. The organizational‐silence branch was not restricted by qualitative‐design terms to avoid missing relevant studies that did not explicitly identify their qualitative design in the indexed fields. The final database‐specific search strategies are presented in Table 1. Grey literature was not searched because the review was restricted to indexed full reports whose participant quotations and contextual descriptions could be traced and appraised consistently.

**TABLE 1:** Database‐specific search strategies.

Database	Search strategy
PsycINFO	(TI nurs* OR AB nurs* OR SU nurs*) AND (((TI (“organizational silence” OR “organizational silence” OR “employee silence” OR “workplace silence” OR “silence behaviour*” OR “silence behaviour*” OR “silent behaviour*” OR “silent behaviour*”)) OR (AB (“organizational silence” OR “organizational silence” OR “employee silence” OR “workplace silence” OR “silence behaviour*” OR “silence behaviour*” OR “silent behaviour*” OR “silent behaviour*”)) OR (SU (“organizational silence” OR “organizational silence” OR “employee silence” OR “workplace silence”))) OR (((TI (“speaking up” OR “speak up” OR “failure to speak up” OR “voice behaviour*” OR “voice behaviour*” OR “employee voice” OR “withholding concern*” OR “withhold* concern*” OR “withhold* information”)) OR (AB (“speaking up” OR “speak up” OR “failure to speak up” OR “voice behaviour*” OR “voice behaviour*” OR “employee voice” OR “withholding concern*” OR “withhold* concern*” OR “withhold* information”))) AND ((TI (qualitative OR interview* OR “focus group*” OR phenomenolog* OR ethnograph* OR “grounded theory” OR “thematic analys*” OR “content analys*” OR “framework analys*” OR “narrative analys*” OR “mixed method*”)) OR (AB (qualitative OR interview* OR “focus group*” OR phenomenolog* OR ethnograph* OR “grounded theory” OR “thematic analys*” OR “content analys*” OR “framework analys*” OR “narrative analys*” OR “mixed method*”)) OR (SU (qualitative OR interview* OR “focus group*” OR phenomenolog* OR ethnograph* OR “grounded theory”)))))
CINAHL	((MH “Nurses+”) OR TI nurs* OR AB nurs* OR TI “nursing personnel” OR AB “nursing personnel” OR TI “registered nurse*” OR AB “registered nurse*” OR TI “clinical nurse*” OR AB “clinical nurse*” OR TI “staff nurse*” OR AB “staff nurse*”) AND (((TI (“organizational silence” OR “organizational silence” OR “employee silence” OR “workplace silence” OR “silence behaviour*” OR “silence behaviour*” OR “silent behaviour*” OR “silent behaviour*”)) OR (AB (“organizational silence” OR “organizational silence” OR “employee silence” OR “workplace silence” OR “silence behaviour*” OR “silence behaviour*” OR “silent behaviour*” OR “silent behaviour*”))) OR (((TI (“speaking up” OR “speak up” OR “failure to speak up” OR “voice behaviour*” OR “voice behaviour*” OR “employee voice” OR “withholding concern*” OR “withhold* concern*” OR “withhold* information”)) OR (AB (“speaking up” OR “speak up” OR “failure to speak up” OR “voice behaviour*” OR “voice behaviour*” OR “employee voice” OR “withholding concern*” OR “withhold* concern*” OR “withhold* information”))) AND ((MH “Qualitative Studies+”) OR (MH “Interviews+”) OR (MH “Focus Groups”) OR TI (qualitative OR interview* OR “focus group*” OR phenomenolog* OR ethnograph* OR “grounded theory” OR “thematic analys*” OR “content analys*” OR “framework analys*” OR “narrative analys*” OR “mixed method*”) OR AB (qualitative OR interview* OR “focus group*” OR phenomenolog* OR ethnograph* OR “grounded theory” OR “thematic analys*” OR “content analys*” OR “framework analys*” OR “narrative analys*” OR “mixed method*”))))
Pubmed	(“Nurses”[Mesh] OR “Nursing Staff, Hospital”[Mesh] OR nurse*[Title/Abstract] OR “nursing personnel”[Title/Abstract] OR “registered nurse*”[Title/Abstract] OR “clinical nurse*”[Title/Abstract] OR “staff nurse*”[Title/Abstract]) AND ((“organizational silence”[Title/Abstract] OR “organizational silence”[Title/Abstract] OR “employee silence”[Title/Abstract] OR “workplace silence”[Title/Abstract] OR “silence behaviour*”[Title/Abstract] OR “silence behaviour*”[Title/Abstract] OR “silent behaviour*”[Title/Abstract] OR “silent behaviour*”[Title/Abstract]) OR ((“speaking up”[Title/Abstract] OR “speak up”[Title/Abstract] OR “failure to speak up”[Title/Abstract] OR “voice behaviour*”[Title/Abstract] OR “voice behaviour*”[Title/Abstract] OR “employee voice”[Title/Abstract] OR “withholding concern*”[Title/Abstract] OR “withhold* concern*”[Title/Abstract] OR “withhold* information”[Title/Abstract]) AND (“Qualitative Research”[Mesh] OR “Interviews as Topic”[Mesh] OR “Focus Groups”[Mesh] OR qualitative[Title/Abstract] OR interview*[Title/Abstract] OR “focus group*”[Title/Abstract] OR phenomenolog*[Title/Abstract] OR ethnograph*[Title/Abstract] OR “grounded theory”[Title/Abstract] OR “thematic analys*”[Title/Abstract] OR “content analys*”[Title/Abstract] OR “framework analys*”[Title/Abstract] OR “narrative analys*”[Title/Abstract] OR “mixed method*”[Title/Abstract])))
Scopus	TITLE‐ABS‐KEY (nurs*) AND (TITLE‐ABS‐KEY (“organizational silence” OR “organizational silence” OR “employee silence” OR “workplace silence” OR “silence behaviour*” OR “silence behaviour*” OR “silent behaviour*” OR “silent behaviour*”) OR (TITLE‐ABS‐KEY (“speaking up” OR “speak up” OR “failure to speak up” OR “voice behaviour*” OR “voice behaviour*” OR “employee voice” OR “withholding concern*” OR “withhold* concern*” OR “withhold* information”) AND TITLE‐ABS‐KEY (qualitative OR interview* OR “focus group*” OR phenomenolog* OR ethnograph* OR “grounded theory” OR “thematic analys*” OR “content analys*” OR “framework analys*” OR “narrative analys*” OR “mixed method*”)))
Embase	(“nurse”/exp. OR nurs*:ti,ab,kw OR “nursing personnel”:ti,ab,kw OR “registered nurse*”:ti,ab,kw OR “clinical nurse*”:ti,ab,kw OR “staff nurse*”:ti,ab,kw) AND ((“organizational silence”:ti,ab,kw OR “organizational silence”:ti,ab,kw OR “employee silence”:ti,ab,kw OR “workplace silence”:ti,ab,kw OR “silence behaviour*”:ti,ab,kw OR “silence behaviour*”:ti,ab,kw OR “silent behaviour*”:ti,ab,kw OR “silent behaviour*”:ti,ab,kw) OR ((“speaking up”:ti,ab,kw OR “speak up”:ti,ab,kw OR “failure to speak up”:ti,ab,kw OR “voice behaviour*”:ti,ab,kw OR “voice behaviour*”:ti,ab,kw OR “employee voice”:ti,ab,kw OR “withholding concern*”:ti,ab,kw OR “withhold* concern*”:ti,ab,kw OR “withhold* information”:ti,ab,kw) AND (“qualitative research”/exp. OR “interview”/exp. OR “focus group”/exp. OR qualitative:ti,ab,kw OR interview*:ti,ab,kw OR “focus group*”:ti,ab,kw OR phenomenolog*:ti,ab,kw OR ethnograph*:ti,ab,kw OR “grounded theory”:ti,ab,kw OR “thematic analys*”:ti,ab,kw OR “content analys*”:ti,ab,kw OR “framework analys*”:ti,ab,kw OR “narrative analys*”:ti,ab,kw OR “mixed method*”:ti,ab,kw)))
CNKI	Topic/keywords (组织沉默 OR 员工沉默 OR 沉默行为 OR 建言 OR 发声) AND (护士 OR 临床护士 OR 护理人员) AND (质性研究 OR 质性访谈 OR 访谈 OR 现象学 OR 扎根理论 OR 主题分析)
Wanfang Data	Topic/keywords (组织沉默 OR 员工沉默 OR 沉默行为 OR 建言 OR 发声) AND (护士 OR 临床护士 OR 护理人员) AND (质性研究 OR 质性访谈 OR 访谈 OR 现象学 OR 扎根理论 OR 主题分析)
VIP	Topic/keywords (组织沉默 OR 员工沉默 OR 沉默行为 OR 建言 OR 发声) AND (护士 OR 临床护士 OR 护理人员) AND (质性研究 OR 质性访谈 OR 访谈 OR 现象学 OR 扎根理论 OR 主题分析)

### 2.4 Study Selection and Supplementary Searching

The first author conducted the database searches, screened titles and abstracts, and assessed potentially eligible full texts against the pre‐specified criteria. Of 1270 records identified, 844 duplicates were removed, leaving 426 records for title and abstract screening. At this stage, 386 records were excluded. The full texts of the remaining 40 reports were assessed, and 21 were excluded for not meeting the eligibility criteria. Nineteen reports were included in the qualitative meta‐synthesis. The selection process is shown in Figure 1, and the characteristics of the included reports are presented in Table 2.

**FIGURE 1:**
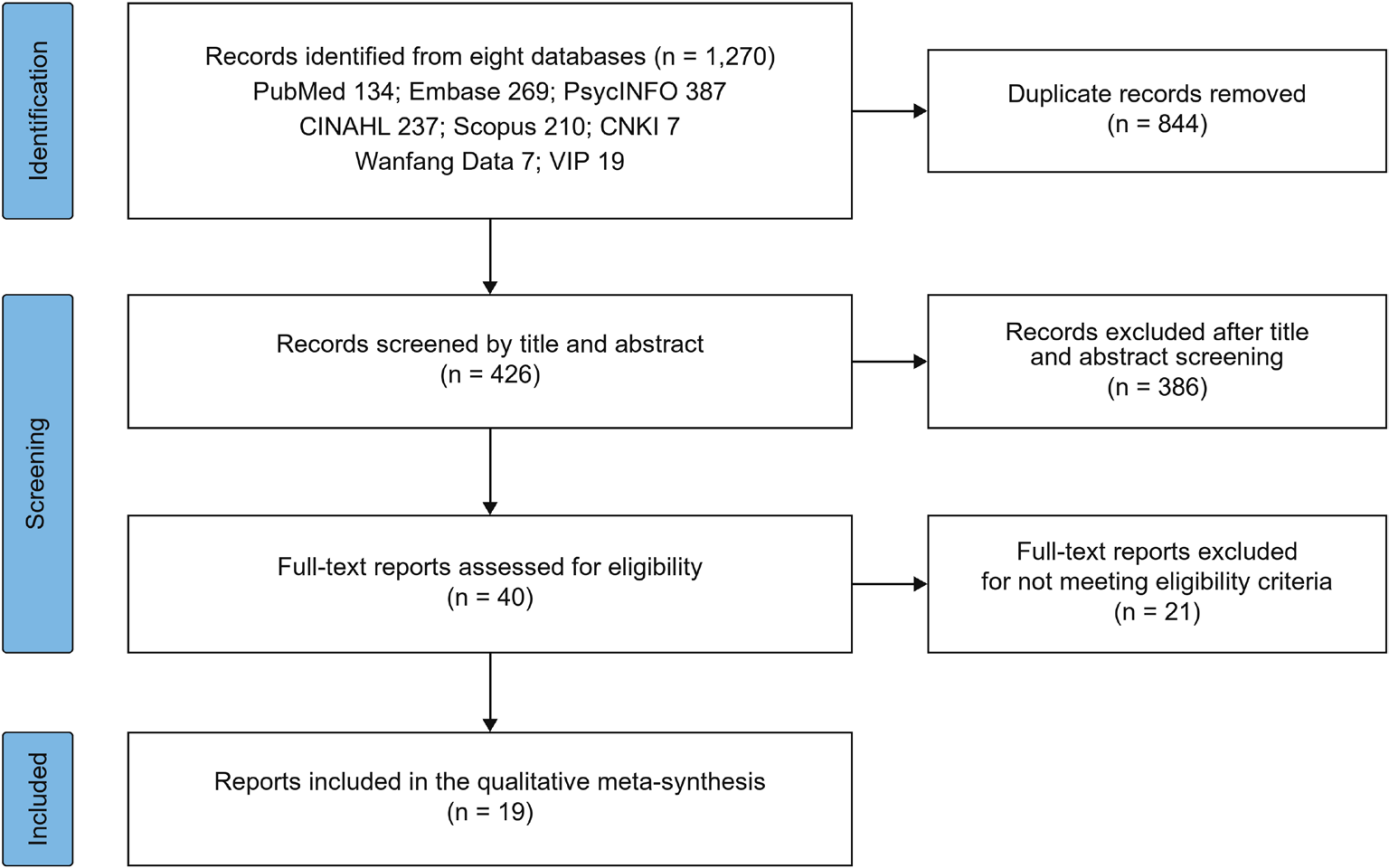
Literature screening process.

**TABLE 2:** Characteristics of the included studies.

Article title	Authors	Country	Language	Year	Study design and analysis	Study aim	Participants	Sample size	Primary‐study themes
The organizational silence experiences of Generation Z nurses in emergency department: a qualitative descriptive phenomenological research study in Shanghai, China	Jiang et al.	China	English	2026	Qualitative descriptive phenomenology; semi‐structured interviews; Colaizzi's seven‐step analysis	To explore organizational silence experiences among Generation Z emergency department nurses in the Chinese sociocultural context.	Generation Z emergency department nurses at a tertiary hospital in Shanghai	21	Competency limitations among those maintaining silence; underlying reasons for organizational silence; outcomes of silence.
A phenomenological study of organizational silence among male emergency department nurses [in Chinese]	Liu et al.	China	Chinese	2021	Interpretive phenomenology; semi‐structured interviews; Van Manen analysis	To examine the lived experience of organizational silence among male emergency department nurses.	Male emergency department nurses	14	Fear and apprehension in silence; awe and obedience in silence; comity and harmony in silence.
Orthopaedic nurses' lived experiences of organizational silence: a qualitative study [in Chinese]	Cheng et al.	China	Chinese	2021	Phenomenology; semi‐structured interviews; thematic analysis	To explore orthopaedic nurses' lived psychological experiences of organizational silence.	Registered nurses providing direct orthopaedic care	16	Negative emotional experiences; diverse reasons for organizational silence; increased burnout; negative leadership behaviours; increased risks to nursing quality and patient safety.
Analysis and interpretation of organizational silence behaviour of male nurses from the perspective of phenomenology	Yan et al.	China	English	2025	Interpretive phenomenology; online semi‐structured interviews; Colaizzi analysis	To examine the underlying reasons for organizational silence among male nurses.	Male clinical nurses at four tertiary hospitals	16	Leadership influence on speaking up; low social acceptance; subjective influences.
Barriers to nurses health advocacy role	Laari and Duma	Ghana	English	2023	Qualitative descriptive study; in‐depth interviews; content analysis	To explore circumstances that prevent nurses from fulfilling their health advocacy role.	Professional nurses and midwives at three regional hospitals	24 overall; numbers by profession not reported	Intrapersonal barriers; interpersonal barriers; structural barriers.
Nurses' experiences of organizational silence: a qualitative study [in Chinese]	Yin et al.	China	Chinese	2016	Phenomenological study; semi‐structured in‐depth interviews	To explore clinical nurses' experiences of organizational silence.	Clinical nursing personnel	12	Negative emotional experiences; lack of organizational belonging; weighing costs and benefits before remaining silent; remaining silent because of insufficient competence.
Motivators and inhibitors of nurses' speaking up behaviours: a descriptive qualitative study	Lee et al.	South Korea	English	2022	Qualitative descriptive study; semi‐structured interviews; inductive content analysis	To identify factors that motivate or inhibit nurses from speaking up for patient safety.	Nurses from four hospitals	15	Motivators: safety culture, supportive managers and role models, positive reactions or familiarity, high‐risk situations, and personal characteristics. Inhibitors: hierarchy, seniority and unit tenure, relationship concerns, and workload.
Silencing behaviour of nurses in the operating theatre [in Chinese]	Qi et al.	China	Chinese	2024	Interpretive phenomenology; semi‐structured interviews; Colaizzi's seven‐step analysis	To explore operating room nurses' experiences of organizational silence and the reasons for it.	Operating room nurses at a tertiary hospital	12	Three themes were identified: the phenomenon of silence, reasons for silence, and coping approaches. Eight subthemes addressed degree and form of silence, psychological vulnerability, fear of isolation, work constraints, forced compliance, seeking support, and leaving the environment.
Experiences of organizational silence among early‐career nurses: a qualitative study [in Chinese]	Wu et al.	China	Chinese	2023	Qualitative interview study; Colaizzi's seven‐step analysis	To explore early‐career nurses' feelings and experiences of organizational silence.	Early‐career registered nurses at a tertiary hospital	12	Worries and concerns about speaking up; lack of organizational belonging; preserving harmony through silence.
Factors influencing newly graduated registered nurses' voice behaviour: an interview study	Kee and de Jong	Netherlands	English	2022	Qualitative descriptive study; semi‐structured interviews	To identify factors affecting newly graduated registered nurses' voice behaviour.	Newly graduated registered nurses working in inpatient hospital settings	17	Self‐confidence; feeling encouraged and welcome to speak up; relationship with the recipient; content of the concern.
Cultural factors influencing Japanese nurses' assertive communication. Part 1: Collectivism	Omura et al.	Japan	English	2018	Semi‐structured interviews; directed content analysis	To examine how collectivist cultural values shape Japanese nurses' assertive communication.	Japanese registered nurses; same participants as report (Yan et al. 2025)	23; not additional to (Yan et al. 2025)	Four categories within collectivism: wa (harmony), uchi to soto (inside and outside), implicit communication and ambiguity, and nemawashi (groundwork).
Influencing factors of organizational silence of nurses: a qualitative study [in Chinese]	Du and Li	China	Chinese	2025	Semi‐structured in‐depth interviews; Colaizzi's seven‐step analysis	To identify factors influencing organizational silence among clinical nurses.	Clinical registered nurses from six tertiary hospitals	20	Three themes—individual, interpersonal, and organizational factors—with seven subthemes concerning experience and competence, ownership, worry and compromise, negative feedback, using an intermediary, organizational complexity, and nurse managers' non‐positional influence.
Exploring the perspectives of operating room nurses about speaking up in a tertiary hospital in Jordan: a qualitative study	Albsoul et al.	Jordan	English	2026	Qualitative descriptive study; individual semi‐structured interviews; inductive thematic analysis	To explore operating room nurses' views on speaking up about patient safety.	Operating room nurses at a tertiary hospital	12	The power of nurses' voice; consequences of speaking up; barriers to speaking up; organizational support for speaking up.
Cultural factors influencing Japanese nurses' assertive communication. Part 2: Hierarchy and power	Omura et al.	Japan	English	2018	Semi‐structured interviews; directed content analysis	To examine how hierarchy and power shape Japanese nurses' assertive communication.	Japanese registered nurses; same participants as report (Jiang et al. 2026)	23; not additional to (Jiang et al. 2026)	Five categories within hierarchy and power: professional status, gender imbalance, seniority and generation gap, bullying, and humility and modesty.
Assertive communication: the Indian nurses' perspective	Chauhan and Tiwari	India	English	2025	Qualitative study; face‐to‐face semi‐structured interviews	To explore Indian nurses' perceptions and use of assertive communication at work.	Hospital nurses in India	7	Obliviousness to assertive communication; vertical dominance; unfavourable climates; readiness for assertive communication; gaining leverage.
Interprofessional model on speaking up behaviour in healthcare professionals: a qualitative study	Umoren et al.	United States	English	2022	Qualitative descriptive study; focus groups and interviews; content analysis	To identify barriers and facilitators to speaking up about patient safety concerns.	Mixed professional sample including nurses, advanced practice providers, and physicians	62 overall; clinical registered nurse subgroup not reported	Two themes based on the mixed professional sample: medical hierarchy impedes speaking up; institutional, interpersonal, and individual factors modify its effects.
Speaking up during the COVID‐19 pandemic: nurses' experiences of organizational disregard and silence	Abrams et al.	United Kingdom	English	2023	Longitudinal qualitative study; repeated remote interviews; thematic analysis	To examine nurses' experiences of speaking up during COVID‐19 and the consequences of doing so.	Clinical nurses and other nursing‐related participants, including a midwife and student nurses	50 overall; eligible clinical nurse subgroup not reported	Under threat: the ability to speak up or not; risk tolerance and avoidance; deafness and hostility in responses to speaking up.
South Korean nurses' experiences of speaking up for patient safety and incident prevention	Jeong and Kim	South Korea	English	2023	In‐depth interviews; inductive content analysis	To explore nurses' experiences of speaking up to prevent patient safety incidents.	Nurses with patient safety or education experience in clinical, quality improvement, and management roles	12; direct‐care subgroup not reported	Current scenario of speaking up; barriers to speaking up; strategies for speaking up; confidence training.
‘Saying no without saying no’: an organizational case study on assertive communication practices among nursing workforce in Saudi Arabia	Mansour et al.	Saudi Arabia	English	2025	Qualitative descriptive case study; four focus groups, four interviews, and document analysis; thematic analysis	To examine sociocultural strategies used to express and respond to assertive communication.	Frontline nurses, nurse managers, physicians, pharmacists, and senior managers	28 overall; 13 frontline nurses	Three themes based on mixed professional data: understanding the cultural context; tactical choices for practicing assertiveness; change on the horizon.

*Note:* (1) Reports Jiang et al. (2026) and Yan et al. (2025) draw on the same 23 nurses. They are separate reports but do not represent independent participant samples. (2) Reports Zou et al. (2025), Yin et al. (2016), Lee et al. (2022), and Wu et al. (2023) include mixed participant groups. Report Qi et al. (2024) includes quality‐improvement and management roles. The synthesis should use only findings clearly attributable to registered nurses providing direct clinical care; the eligibility of report Qi et al. (2024) requires particular review. (3) Overall sample size should not be interpreted as the number of participants eligible for this review when the primary report did not provide a separate count.

### 2.5 Eligibility Criteria

Studies were included if they met all four criteria:

1. Population. Participants were registered nurses engaged in clinical care, or nurses with equivalent professional qualifications in their countries. In mixed‐profession reports, the clinical nurses' findings had to be separately extractable.
2. Phenomenon of interest. The report provided qualitative findings on withholding work‐related concerns, information, suggestions, or patient‐safety concerns. Studies of speaking up, patient advocacy, or assertive communication were included only when they described barriers to expression or decisions not to speak up.
3. Study design. A qualitative design was used, or an independently extractable qualitative component was reported in a mixed‐methods study.
4. Report availability. A Chinese‐ or English‐language full report was available.

Reports were excluded for any of the following reasons:

1. The sample comprised only students, nursing assistants, nonclinical personnel, or managers who did not provide direct clinical care, or the clinical nurses' findings could not be separated from a mixed sample.
2. No extractable qualitative finding concerned withholding or barriers to expression, or the study used quantitative methods only.
3. The publication was a review, commentary, or study protocol, or duplicated findings from another report of the same study.
4. The full text could not be obtained.

### 2.6 Data Extraction and Synthesis

Title and abstract screening and full‐text eligibility assessment were performed by the first author alone. Following study selection, two postgraduate nursing students from the same research group undertook data extraction and verification: one extracted study characteristics, participant quotations, and primary‐study authors' interpretations, and the other checked these data against the original reports. Study characteristics were extracted from the methods and results sections, and qualitative evidence from the results or findings sections. Both students independently coded the extracted evidence and assessed methodological quality. Discrepancies in extraction, coding, or appraisal were resolved through discussion, with a third research‐group member involved until consensus was reached. Chinese‐language evidence was coded in Chinese, and all quotations translated into English were checked against the original source texts before manuscript finalization. The team recognized that shared nursing training and research‐group membership could lead to similar assumptions about what constituted organizational silence. To examine these assumptions, participant accounts were distinguished from primary‐study interpretations; accounts challenging emerging categories were retained, and analytical claims were checked against the source evidence and study context.

Following Thomas and Harden's thematic synthesis approach, the extracted qualitative data were organized and analysed using NVivo 15.0. Participant quotations and related findings were coded line by line and grouped into descriptive themes through constant comparison. Analytical themes were then developed across studies while retaining differences in motivation, context, and behavioural expression. Coding was inductive and was not guided by predetermined silence categories. The resulting themes were subsequently examined in relation to the four silence patterns discussed in the synthesis.

Social information processing theory was applied after theme development to interpret how earlier interactions and organizational responses might inform later judgements about expressing concerns. It did not determine the initial codes or the identification of silence types. Participant quotations were treated as first‐order evidence, primary‐study authors' interpretations as second‐order evidence, and the review‐team themes as third‐order interpretations. All interpretations were checked against the original quotations, participant identifiers, and study contexts, with contradictory accounts retained.

### 2.7 Methodological Quality Appraisal

After eligibility had been determined, two reviewers independently assessed all included reports using the 10‐item Joanna Briggs Institute Critical Appraisal Checklist for Qualitative Research. Items were rated Yes, No, Unclear, or Not applicable. Disagreements were discussed with a third research‐group member until consensus was reached. Appraisal informed interpretation of the evidence but was not used to exclude reports. No summary score or quality cutoff was applied.

All 19 eligible reports were retained. The item‐level appraisal matrix in Table 3 shows variation in reporting of researcher positioning, reflexivity, and ethical procedures. These judgements were considered when interpreting the synthesis but did not affect eligibility.

**TABLE 3:** Detailed methodological quality appraisal results.

Author (year)	Q1	Q2	Q3	Q4	Q5	Q6	Q7	Q8	Q9	Q10
Jiang et al. (2026)	Yes	Yes	Yes	Yes	Yes	No	Unclear	Yes	Yes	Yes
Liu et al. (2021)	Yes	Yes	Yes	Yes	Yes	Yes	Unclear	Yes	Yes	Yes
Cheng et al. (2021)	Unclear	Yes	Yes	Yes	Yes	No	No	Yes	No	Yes
Yan et al. (2025)	Yes	Yes	Yes	Yes	Yes	No	No	Yes	Yes	Yes
Laari and Duma (2023)	Unclear	Yes	Yes	Yes	Yes	No	No	Yes	Yes	Yes
Yin et al. (2016)	Unclear	Yes	Yes	Unclear	Yes	No	Unclear	Yes	No	Yes
Lee et al. (2022)	Unclear	Yes	Yes	Yes	Yes	Unclear	Unclear	Yes	Yes	Yes
Qi et al. (2024)	No	Yes	Yes	Yes	Yes	No	No	Yes	Yes	Yes
Wu et al. (2023)	Unclear	Yes	Yes	Yes	Yes	No	No	Yes	No	Yes
Kee and de Jong (2022)	Unclear	Yes	Yes	Yes	Yes	No	Unclear	Yes	Yes	Yes
Omura et al. (2018a)	Unclear	Yes	Yes	Yes	Yes	Yes	Yes	Yes	Yes	Yes
Du and Li (2025)	Unclear	Yes	Yes	Yes	Yes	No	No	Yes	Yes	Yes
Albsoul et al. (2026)	Unclear	Yes	Yes	Yes	Yes	Unclear	Unclear	Yes	Yes	Yes
Omura et al. (2018b)	Unclear	Yes	Yes	Yes	Yes	No	No	Yes	Yes	Yes
Chauhan and Tiwari (2025)	Unclear	Yes	Yes	Yes	Yes	No	No	Yes	Yes	Yes
Umoren et al. (2022)	Unclear	Yes	Yes	Yes	Yes	Unclear	No	Yes	Yes	Yes
Abrams et al. (2023)	Yes	Yes	Yes	Yes	Yes	Yes	Unclear	Yes	Yes	Yes
Jeong and Kim (2023)	Unclear	Yes	Yes	Yes	Yes	No	No	Yes	Yes	Yes
Mansour et al. (2025)	Unclear	Yes	Yes	Yes	Yes	Yes	Unclear	Yes	Yes	Yes

JBI criteria: Q1, congruity between the stated philosophical perspective and methodology; Q2, congruity between methodology and the research question or objectives; Q3, congruity between methodology and data collection; Q4, congruity between methodology and the representation and analysis of data; Q5, congruity between methodology and the interpretation of results. Q6, statement locating the researcher culturally or theoretically; Q7, influence of the researcher on the research, and vice versa, addressed; Q8, participants and their voices adequately represented; Q9, ethical approval and appropriate ethical conduct reported; Q10, conclusions flow from the analysis or interpretation of the data. Ratings: Yes, No, Unclear, or Not applicable.

## 3 Results

Comparison of participants' accounts with the interpretations reported by the original study authors generated four analytical themes comprising 12 review findings. These themes concerned the motives underlying organizational silence, how participants linked earlier organizational responses to later decisions to speak or remain silent, the distinction between withholding and alternative forms of expression, and the organizational conditions that constrained communication. Each subtheme was treated as a separate review finding for the GRADE‐CERQual assessment. The GRADE‐CERQual confidence assessments and explanations for all 12 review findings are presented in Table S2.

### 3.1 Motives Distinguished Forms of Organizational Silence

#### 3.1.1 Anticipated Adverse Consequences

Nurses withheld concerns when they expected an unfavourable response from managers or colleagues. A Generation Z emergency department nurse said, ‘I'm worried about retaliation (from management)’ (Jiang et al. 2026). Male emergency department nurses described holding back unfavourable information when reporting to leaders because their words might be misunderstood (Liu et al. 2021). In an orthopaedic unit, one nurse hesitated to express her genuine views during departmental discussions because they might differ from the leader's views. Another described avoiding substantive issues to preserve relationships with colleagues and avoid offending the leader (Cheng et al. 2021). These accounts describe anticipated consequences ranging from misunderstanding and strained working relationships to retaliation. The orthopaedic study did not report that disagreement itself led to punishment.

Other accounts concerned professional reputation and relationships. A male nurse avoided commenting on colleagues' work for fear of becoming a target of criticism (Yan et al. 2025). A nurse who had been negatively labelled after advocating for patients expected further trouble if she raised another concern (Laari and Duma 2023). During the COVID‐19 pandemic, a nurse described feeling constrained after her manager invoked the professional code of conduct in response to her objection (Abrams et al. 2023). Although the anticipated consequences differed, these accounts were consistent with defensive organizational silence.

#### 3.1.2 Expected Futility After Limited Influence or Repeated Rejection

Some nurses withheld opinions because they expected little to result from expressing them. One participant questioned whether her view would be heard because she was a staff nurse (Yin et al. 2016). An orthopaedic nurse described no longer proposing suggestions after repeated rejection (Cheng et al. 2021). Male nurses similarly recounted losing hope when earlier attempts had produced no change and saw little value in raising the same proposal again (Yan et al. 2025).

Perceived futility arose in more than one way. For some nurses, limited standing within the unit made their opinions seem unlikely to carry weight. For others, the expectation followed unsuccessful attempts to contribute. The common feature was a reduced expectation that expression would affect a decision or practice, consistent with acquiescent organizational silence.

#### 3.1.3 Protecting Working Relationships

Nurses also considered the relationships on which clinical work depended. Orthopaedic nurses described withholding or redirecting information while considering a colleague's relationship with them and a leader's standing (Cheng et al. 2021). Emergency department nurses in Shanghai referred to team harmony and the need to continue working closely with colleagues (Jiang et al. 2026). South Korean nurses described reluctance to raise a concern when the exchange could damage a relationship needed for subsequent work (Lee et al. 2022). A male nurse likewise avoided offending senior colleagues with whom he had established working relationships (Yan et al. 2025).

These accounts did not all describe the same motive. An intensive care nurse in another South Korean study said that raising another person's mistake could be regarded as meddling (Jeong and Kim 2023). Her account concerned the anticipated interpretation of an intervention as well as the underlying safety issue. Across the studies, withholding could serve a wish to protect cooperation, a concern about the nurse's own position, or both. Relationship preservation alone was therefore insufficient to classify an account as prosocial organizational silence.

### 3.2 Earlier Responses Informed Later Expression and Participation

#### 3.2.1 Dismissal or Criticism Preceded Reduced Expression

Nurses related later withholding to the reception of earlier concerns. An operating room nurse described suggestions being repeatedly rejected and then criticized privately by a leader; staff subsequently became less willing to contribute (Qi et al. 2024). An orthopaedic nurse gradually stopped offering opinions after repeated rejection (Cheng et al. 2021), while another participant described becoming accustomed to remaining silent when her views were not accepted (Yin et al. 2016). After her ideas were dismissed, an emergency department nurse chose to listen more and speak less to avoid ridicule (Jiang et al. 2026).

In a study of male nurses, objections to unfair scheduling were not addressed, and those who raised them were treated as troublemakers; participants described subsequently withholding concerns (Yan et al. 2025). A children's nurse who had emailed a concern without receiving a meaningful response recalled, ‘I remember thinking that was pointless me even sending that email out’ (Abrams et al. 2023). These accounts linked an ineffective response with reduced expectations of change. Where dismissal was accompanied by criticism of the person speaking, the same encounter also informed expectations about the personal consequences of another attempt. The findings indicate a possible connection between acquiescent and defensive motives, rather than a fixed sequence between them.

#### 3.2.2 Constructive Responses Supported Continued Expression

Accounts of attentive responses provided a contrast. An early‐career nurse whose suggestion was accepted by a head nurse described being willing to propose further improvements (Wu et al. 2023). South Korean nurses linked managers' listening and action, a physician's acknowledgment and correction of a problem, and a manager's explanation when an issue could not be resolved with greater willingness to raise later concerns (Lee et al. 2022). Newly graduated nurses also described being more willing to express concerns when their views were heard and taken seriously (Kee and de Jong 2022).

A constructive response did not always require immediate adoption of a suggestion. In these accounts, acknowledgment, an explanation of the decision, or action on the concern gave nurses information about how their contribution had been received. These experiences differed from repeated rejection or an absent response in the expectations nurses described for later expression.

#### 3.2.3 Reduced Involvement Had Different Temporal Positions

Some nurses described reduced participation after a period of silence or unsuccessful expression. Orthopaedic nurses associated prolonged organizational silence with declining enthusiasm for departmental affairs and a preference to concentrate on assigned duties (Cheng et al. 2021). A male nurse reported becoming less concerned about work after repeated concerns about workload and inadequate rest brought no improvement (Yan et al. 2025).

Other accounts placed limited involvement earlier in the sequence. Emergency department nurses who expected to leave the unit or regarded organizational matters as outside their immediate duties described little reason to intervene (Jiang et al. 2026). An early‐career nurse in a rotational placement similarly felt less connected to the unit's affairs (Wu et al. 2023). Reduced participation appeared both after unsuccessful engagement and alongside a limited attachment to the unit. Participation level alone did not identify a particular motive for organizational silence.

### 3.3 Communication Route and Timing Shaped the Distinction Between Expression and Withholding

#### 3.3.1 Changing the Recipient or Route of Expression

Nurses adjusted communication according to the person they needed to address. Japanese nurses found established colleagues easier to approach than unfamiliar or higher‐ranking physicians and sometimes sought individual views before raising an issue more widely (Omura et al. 2018a). A South Korean nurse was more comfortable addressing a familiar colleague who was likely to understand a reminder as concern rather than criticism (Lee et al. 2022). In a Chinese study, nurses sometimes asked a colleague with a better relationship with the head nurse to convey a suggestion (Du and Li 2025).

Access to a helpful colleague also mattered in a Saudi Arabian case study. A non‐Saudi frontline nurse described greater difficulty during evening shifts, when fewer Saudi nursing colleagues were available to help with challenging interactions (Mansour et al. 2025). Her account identified a constraint on communication but did not establish that a particular concern was conveyed or withheld. By contrast, where a colleague passed on the substantive issue or a nurse later raised it herself, the account described an adapted form of expression. Jordanian operating room nurses described a different situation: some avoided commenting to surgeons whom they expected to respond negatively, leaving the concern unexpressed (Albsoul et al. 2026).

#### 3.3.2 Deferring Nonurgent Concerns

Operating room nurses distinguished observations needing immediate action from matters that could be discussed after a procedure. Some reported raising nonurgent issues at the end of surgery, partly because disagreement in front of a patient could undermine confidence in the team (Albsoul et al. 2026). These accounts described a change in timing when later communication was explicitly reported.

The later course of a concern was not always clear. A South Korean nurse refrained from addressing an intern's protective practice while the intern was busy, but the account did not state that she raised the matter afterward (Lee et al. 2022). It documents withholding at that time without establishing its duration. Evidence of deferred expression therefore rests on reported later communication, not simply on the absence of an immediate objection.

#### 3.3.3 Raising Urgent Safety Concerns Despite Interpersonal Risk

The urgency of a patient safety concern could change a nurse's expression choice. South Korean nurses described speaking despite possible interpersonal consequences when immediate patient harm was at stake and regarded withholding a serious safety concern as potentially harmful (Lee et al. 2022). In a Jordanian operating room, one nurse described directly addressing a breach of sterile practice as part of her clinical responsibility (Albsoul et al. 2026).

Urgency did not produce the same response in every encounter. Another Jordanian nurse reported avoiding correction of people who resisted it, despite recognizing the possible consequences for sterile practice (Albsoul et al. 2026). These contrasting accounts show that perceived patient risk could favour direct expression while the expected response from the recipient could still inhibit it.

### 3.4 Organizational Conditions Constrained Expression Across Motives

#### 3.4.1 Professional Hierarchy and Position Within the Unit

Professional hierarchy affected nurses' expectations of being heard and their perceived authority to question others. Japanese nurses identified hierarchy and established reporting order as sources of hesitation (Omura et al. 2018b). An orthopaedic nurse retained her view when a problem was handled primarily under physicians' authority (Cheng et al. 2021). Indian nurses questioned whether their knowledge or qualifications were sufficient to challenge physicians (Chauhan and Tiwari 2025). In an interprofessional study, an RN who described herself as new to practice said that uncertainty about her knowledge made it difficult to challenge a physician's judgement (Umoren et al. 2022). This last account is attributable to a nurse; the study's themes derived from the full interprofessional sample were not treated as nurse‐specific findings.

Position within a unit also changed nurses' access to discussion. Newly graduated nurses described waiting to participate while they learned the local working environment (Kee and de Jong 2022). An early‐career nurse who did not feel accepted as a unit member preferred to observe a problem rather than raise it (Wu et al. 2023). By contrast, a South Korean nurse described speaking more freely after gaining experience and a stronger sense of standing within the unit (Lee et al. 2022). Hierarchy and unit position shaped the circumstances in which concerns were withheld without constituting separate silence motives.

#### 3.4.2 Workload Limited Opportunities to Raise Concerns

Clinical demands reduced the time and energy available to formulate and communicate concerns. A South Korean nurse described having insufficient time for basic nursing care, let alone speaking up (Lee et al. 2022). Jordanian operating room nurses identified workload and patient volume as barriers to expression (Albsoul et al. 2026). An emergency department nurse in Shanghai described withholding views while exhausted by the care of critically ill patients and unwilling to take on further difficulty (Jiang et al. 2026). A male nurse associated high pressure, limited rest, and poor working conditions with diminished willingness to express himself (Yan et al. 2025).

Workload appeared both as an immediate restriction on opportunities to communicate and as part of nurses' assessment of what raising an issue would require of them. The accounts varied in how much they revealed about the nurse's motive; limited expression during a demanding shift did not, on its own, demonstrate resignation or disengagement.

#### 3.4.3 Reporting Routes and Follow‐Up Were Not Always Accessible

Having an idea or concern did not guarantee a usable route for conveying it. An emergency department nurse reported having ideas but no avenue through which to express them (Jiang et al. 2026). In another Chinese study, participants described suggestions passing through a team leader before reaching the head nurse and questioned whether their immediate leaders could resolve problems involving several units (Du and Li 2025). Japanese nurses described a reporting order governed by physicians' rank, including criticism when a nurse approached a senior physician directly (Omura et al. 2018b).

These accounts located constraints at different points. Some affected the nurse's initial opportunity to raise a concern; others affected its route through the organization or the likelihood of follow‐up. When a nurse had already communicated a concern but the organization did not pass it onward or address it, that failure was distinct from the nurse withholding the concern.

## 4 Discussion

Comparison of participant accounts and primary‐study interpretations yielded four analytical themes and 12 subthemes concerning motives for organizational silence, earlier organizational responses and subsequent participation, alternative forms of expression, and organizational constraints on communication. Social information processing theory (Salancik and Pfeffer 1978) was applied after inductive theme development to interpret how workplace cues and prior experiences may shape nurses' expectations about the risks and effectiveness of speaking up. Workload and communication channels were considered practical constraints on expression. The interpretive framework is presented in Figure 2.

**FIGURE 2:**
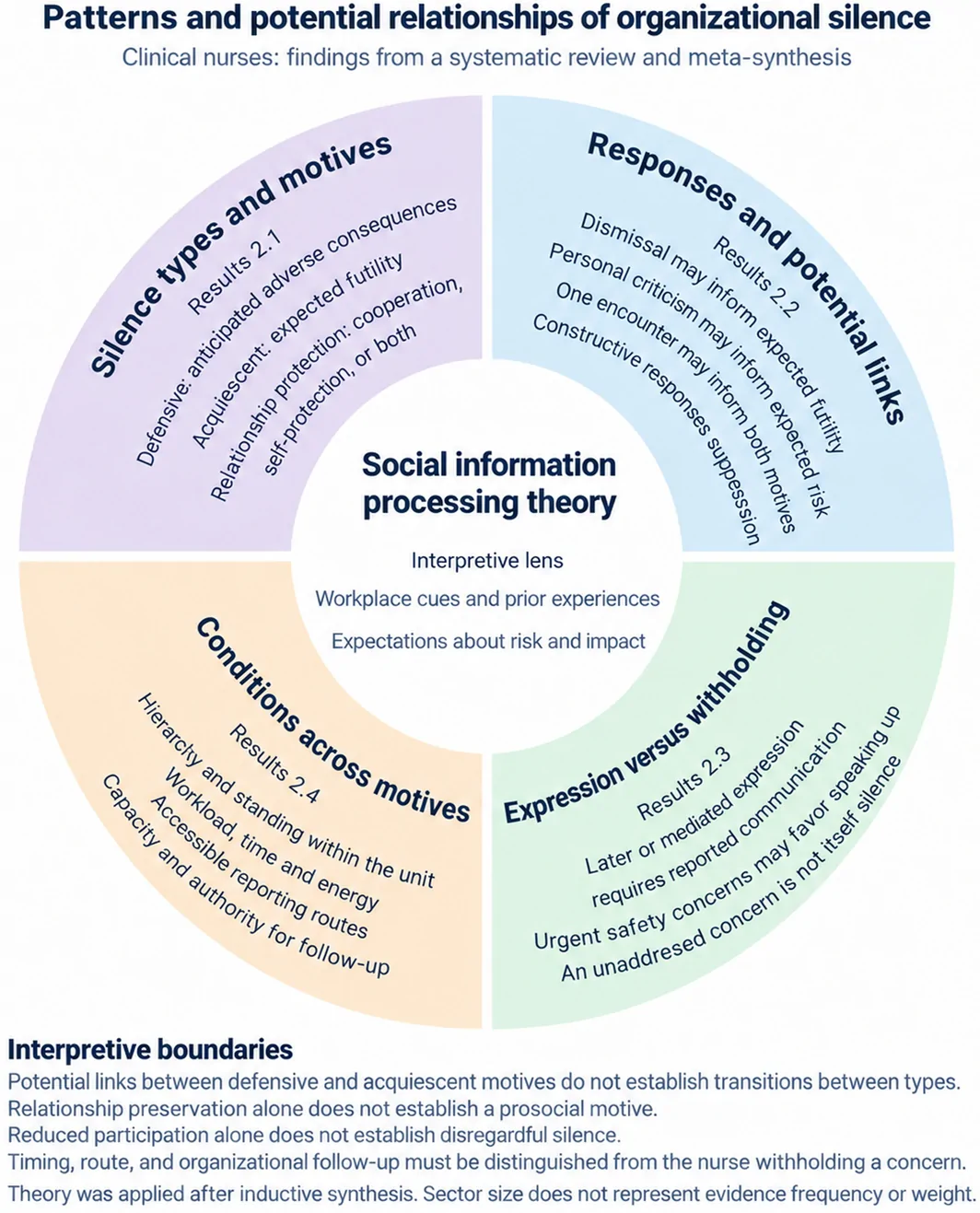
An interpretive framework informed by social information processing theory.

The present review extends the qualitative meta‐synthesis by Zou et al. (2025), which examined nurses' characteristics, silence motives, and consequences, by focusing on relationships among motives, organizational responses, and subsequent communication choices. Its contribution is to refine the interpretation of established silence categories. Dismissal of a concern accompanied by criticism of the nurse may inform both expected futility and perceived personal risk, suggesting a shared context for acquiescent and defensive motives without establishing transitions between them. Relationship preservation alone does not establish a prosocial motive because it may reflect concern for others, self‐protection, or both. Likewise, reduced participation alone does not establish disregardful silence, a category previously identified by Zou et al. (2025). Where later or mediated communication is explicitly reported, it should be distinguished from continued withholding. These distinctions clarify the boundaries of organizational silence while retaining uncertainty where the primary evidence does not establish motives or subsequent communication.

### 4.1 Motives Underlying Different Types of Organizational Silence and Their Shared Contexts

Van Dyne et al. (2003) distinguished defensive, acquiescent, and prosocial organizational silence according to the motive for withholding information. Defensive organizational silence arises from concern about adverse consequences of speaking up. Acquiescent organizational silence reflects the belief that speaking up is unlikely to change the situation. In the studies synthesized here, some nurses described concerns that went unaddressed while those who raised them were regarded as troublemakers. Such experiences could leave nurses concerned about being blamed again and doubtful that another attempt would lead to action. Social information processing theory helps explain how these judgements may arise from the same experience. Nurses observe both what happens to a suggestion and how the person who raised it is treated. Defensive and acquiescent organizational silence remain distinct in motive, even when they arise in a similar context.

In a survey of clinical nurses, Lee et al. (2024) research found a stronger association of psychological safety with organizational silence than with speaking up, whereas perceived impact was more strongly associated with speaking up. This provides indirect support for distinguishing concerns about the consequences of speaking up from expectations about its effectiveness. The survey did not measure specific motives for organizational silence. The accounts in the present synthesis suggest that a single negative experience may inform both judgements. They do not, however, establish that one type of organizational silence develops into another.

Prosocial organizational silence likewise requires attention to the circumstances in which information is withheld. A nurse who avoids a public disagreement may be trying to protect a colleague or preserve teamwork, but may also fear exclusion. A previous qualitative synthesis identified prosocial and defensive organizational silence among nurses (Zou et al. 2025). The present synthesis indicates that accounts of preserving relationships may involve concern for different people. In a survey of clinical nurses, Li et al. (2024) research found associations between abusive supervision, impression management motives, and withholding patient safety concerns. That finding is consistent with self‐protection entering nurses' judgements about relationships, but it does not mean that all relationship‐related organizational silence is self‐protective. From a social information processing perspective, responses from leaders and colleagues help nurses judge how an objection is likely to be received. Where raising a concern is treated as an affront, preserving cooperation and protecting one's own position may both influence the decision to withhold it. Avoiding conflict alone is therefore insufficient to identify a prosocial motive.

Disregardful organizational silence refers to withholding views because of diminished attachment to or identification with one's work or organization (Hu et al. 2025). Reduced participation in unit affairs, however, is not sufficient evidence of this motive. Shen et al. (2025) reported a negative association between work engagement and overall organizational silence scores, but that association does not establish that reduced participation reflects disregardful organizational silence. Repeatedly receiving no response may lower a nurse's expectation that involvement in unit affairs is worthwhile. The accounts synthesized here do not show that every nurse who became less involved deliberately withheld specific information out of indifference. Identifying disregardful organizational silence requires evidence about the concern withheld and the nurse's reason for withholding it.

### 4.2 Organizational Responses and Subsequent Decisions to Speak Up

After repeated dismissal of their suggestions or criticism of those who raised them, some nurses described speaking up less often. Others expressed a willingness to raise further concerns when they had been listened to or given an explanation, even when their suggestions could not be implemented immediately. In social information processing terms, these responses provide cues about how seriously concerns are taken and what nurses might expect from another attempt to speak up.

Lee and Dahinten (2021) research found associations between inclusive leadership, nurses' speaking up and organizational silence, with psychological safety playing a mediating role. In a mixed‐profession healthcare sample, O'Donovan et al. (2021) research found that earlier experiences continued to shape perceptions of the speaking‐up environment after leaders began to show greater support. This offers a basis for understanding why a nurse whose concerns have repeatedly been ignored may remain cautious despite a later, more attentive response. Nurses interpret a new response in light of earlier interactions.

What happens after a concern is raised also depends on organizational arrangements. Jones et al. (2022) research found that limited staff time, resources, and implementation capacity constrained follow‐up within speaking‐up support systems in English healthcare organizations. A constructive response therefore involves more than a receptive manner. Explaining a decision, reporting progress, and acting on a concern require staff capacity and procedures for follow‐up.

### 4.3 Expression Routes, Organizational Conditions, and the Identification of Organizational Silence

Some nurses in the included studies did not raise an objection at the time it arose but later communicated the concern privately, after the immediate task, or through a colleague. Changing the route or timing of expression is not the same as continuing to withhold the information. Lainidi et al. (2023) research noted variation in how healthcare research defines and measures speaking up and organizational silence, and observed that the two may occur in parallel. The present findings indicate that an account should not be classified as organizational silence solely because a nurse did not speak in the original setting or because an expressed concern was not subsequently addressed.

Social information processing theory helps explain how nurses select a recipient and manner of expression. Previous reactions from the intended recipient can inform expectations about the interpersonal consequences of raising an objection. In an experimental study, Weiss et al. (2023) research found that healthcare professionals in teams with higher psychological safety were more likely to view a nurse's suggestion as helpful to team decision‐making. This provides a point of comparison for nurses' concern about how others may evaluate an objection. In a mixed‐profession rehabilitation survey, Niederhauser and Schwappach (2022) research found that judgements about potential patient harm were associated with intentions to speak up, while hierarchical position was associated with responses in some scenarios. In the present synthesis, nurses sometimes deferred nonurgent matters but raised immediate patient safety concerns directly. Professional hierarchy and nurses' standing within a unit shaped their options for expression, while the urgency of the clinical concern also informed its timing.

## 5 Limitations

The proposed relationships among silence types were derived from an interpretive synthesis of experiences reported across separate qualitative studies rather than from longitudinal observations of the same nurses. Moreover, most of the supporting quantitative studies used cross‐sectional designs and therefore could not establish temporal ordering, directionality, or causality. The proposed relationships should consequently be regarded as theoretically informed possibilities rather than a confirmed developmental sequence.

Fifteen of the 19 included reports were conducted in Asian settings, so these contexts are more strongly represented in the synthesis. The reports included nurses from different specialties, career stages, and gender groups, although the number of independent samples in each subgroup did not support a detailed comparison. The review was limited to Chinese‐ and English‐language full reports and did not search grey literature. The review was limited to Chinese‐ and English‐language full reports and did not search grey literature. Relevant qualitative evidence reported in other languages or in sources such as dissertations and institutional reports may therefore have been missed. Primary reports also varied in the detail provided about researcher reflexivity and ethical procedures. These features should be considered when applying the findings in other healthcare settings. Longitudinal and mixed‐methods research could further examine how motives for organizational silence and choices about speaking up vary across settings and over time.

## 6 Implications for Nursing Practice

Nurses may withhold concerns because they anticipate criticism, have previously raised issues without a response, or wish to preserve working relationships. A uniform focus on communication skills is unlikely to address these different circumstances. Clinical supervision and case debriefings can examine the concern, its urgency, the nurse's reasons for holding back, and any communication that followed. When a nurse later raised the issue privately or through another route, timely communication is the relevant consideration. Not speaking publicly at the time should not, by itself, be treated as organizational silence.

Escalation pathways for patient safety concerns need to be usable in everyday clinical work. Unit procedures should identify risks that require immediate escalation and specify the next point of contact when the initial recipient lacks authority to act or does not respond. Procedures and handoffs can limit both the time available and the people a nurse can approach. Nonurgent matters may be discussed after the immediate task, whereas urgent safety concerns require prompt communication. Escalation procedures should also account for night shifts, staffing shortages, and interprofessional care.

Nurse managers and senior clinicians should respond to the substance of a concern. When a suggestion cannot be adopted, explaining the decision and any next steps helps the nurse understand what happened to it. Concerns requiring investigation or coordination across departments also warrant updates as they progress. Nurses may not expect every suggestion to be implemented, but repeated attempts to raise an issue without an explanation may discourage further expression. Teams also need time for handoffs, debriefings, and routine discussion. A reporting channel alone does not create an opportunity to use it during a demanding shift.

Implementation will vary with staffing, professional roles, and existing reporting systems. An increase in reports alone does not demonstrate a reduction in organizational silence. Evaluation should also draw on nurses' accounts of the consequences they anticipated and the responses they received, alongside records of timely communication, follow‐up, and feedback. Together, these sources can show where concerns remain unspoken or encounter difficulties after they have been raised.

## Author Contributions

**Honghu Chen:** writing – original draft, methodology, data curation, resources, conceptualization, investigation, validation, visualization, software, formal analysis, project administration, supervision. **Xuan Cheng:** writing – original draft, formal analysis. **Jiayu Liao:** methodology. **Jiayu Hou:** conceptualization. **Weihong Wang:** writing – review and editing.

## Funding

The authors have nothing to report.

## Ethics Statement

The authors have nothing to report.

## Consent

The authors have nothing to report.

## Conflicts of Interest

The authors declare no conflicts of interest.

## Supporting information


**Table S1:** Reporting checklist.

**Table S2:** GRADE‐CERQual confidence assessments.

## Data Availability

The data that support the findings of this study are available from the corresponding author upon reasonable request.
