# Supplementary material for: Patterns and Potential Relationships of Organizational Silence Types Among Clinical Nurses: A Qualitative Meta‐Synthesis and Systematic Review
Source: Nurs Open. 2026 Nov 3;13(11):e71004. doi: 10.1002/nop2.71004 (PMC13731733; doi:10.1002/nop2.71004)
Supplement: Supplementary file 1 [file NOP2-13-e71004-s001.docx]

| **Results** | **Summary of review finding** | **Contributing reports** | **Confidence / explanation** |
| --- | --- | --- | --- |
| 2.1.1 | Nurses sometimes withheld concerns when they anticipated retaliation, criticism, misunderstanding or damage to their professional position and relationships. | Jiang 2026; Liu 2021; Cheng 2021; Yan 2025; Laari & Duma 2023; Abrams 2023 | Moderate Moderate methodological concerns; otherwise limited concerns for this descriptive finding. |
| 2.1.2 | Some nurses expected their views to have little influence because of limited standing or previous rejection, and consequently described withholding further suggestions. | Yin 2016; Cheng 2021; Yan 2025 | Moderate Moderate methodological concerns and some limitations in the depth and contextual range of the contributing accounts. |
| 2.1.3 | Nurses sometimes withheld or redirected concerns to protect working relationships; these accounts could involve concern for cooperation, self-protection, or both. | Cheng 2021; Jiang 2026; Lee 2022; Yan 2025; Jeong & Kim 2023 (role verification required) | Moderate Moderate methodological concerns; uncertainty about the eligibility of one contributor and the interpretation of motives. |
| 2.2.1 | Nurses described withholding further concerns after earlier suggestions had been ignored, rejected or accompanied by criticism; some accounts combined reduced expectations of influence with fear of personal consequences. | Qi 2024; Cheng 2021; Yin 2016; Jiang 2026; Yan 2025; Abrams 2023 | Moderate Moderate methodological concerns; temporal links are participant accounts rather than demonstrated causal transitions. |
| 2.2.2 | Nurses described greater willingness to raise later concerns when earlier contributions were acknowledged, explained or acted upon; immediate adoption of a suggestion was not always necessary. | Wu 2023; Lee 2022; Kee/de Jong 2022 | Moderate Moderate methodological concerns; consistent first-order support for the stated relationship. |
| 2.2.3 | Reduced involvement in unit affairs was described both after unsuccessful engagement and alongside limited attachment to a unit; participation level alone did not identify a particular silence motive. | Cheng 2021; Yan 2025; Jiang 2026; Wu 2023 | Low Moderate methodological, coherence and adequacy concerns, particularly for the cross-study interpretation of temporal positions. |
| 2.3.1 | Some nurses used familiar colleagues, advance discussion or a colleague intermediary to convey concerns. An adapted route constituted expression only when communication of the substantive concern was reported. | Omura 2018 Part 1; Lee 2022; Du 2025; Mansour 2025; Albsoul 2026 | Moderate Moderate methodological concerns and limited detail on actual transmission in some accounts; explicit examples support the bounded finding. |
| 2.3.2 | A Jordanian operating-room nurse described raising nonurgent concerns after surgery. Withholding at a busy moment alone did not establish that later expression occurred. | Albsoul 2026 (positive evidence); Lee 2022 (boundary account) | Low Serious adequacy concerns for actual deferred expression, with methodological concerns and restricted contextual relevance. |
| 2.3.3 | Some nurses described speaking up when immediate patient safety was at stake despite interpersonal risk, while other accounts showed that an anticipated negative response could still inhibit expression. | Lee 2022; Albsoul 2026 | Low Moderate methodological and adequacy concerns; contrasting accounts support a conditional finding but provide limited explanatory depth. |
| 2.4.1 | Professional hierarchy, seniority and newcomers’ position in a unit shaped nurses’ perceived authority and opportunities to question others; these conditions did not in themselves constitute separate silence motives. | Omura 2018 Part 2; Cheng 2021; Chauhan 2025; Umoren 2022; Kee/de Jong 2022; Wu 2023; Lee 2022 | Moderate Moderate methodological concerns; broad, directly attributed support across several clinical contexts. |
| 2.4.2 | Nurses described workload, fatigue and insufficient time as constraints on formulating or communicating concerns, sometimes alongside concern that raising an issue would create further work. | Lee 2022; Albsoul 2026; Jiang 2026; Yan 2025 | Moderate Moderate methodological concerns; consistent and contextually relevant accounts with some ambiguity about motive. |
| 2.4.3 | Nurses described unavailable channels, rank-governed reporting routes and limited authority to resolve cross-unit issues. A concern already communicated but not forwarded or acted upon was distinct from a nurse withholding it. | Jiang 2026; Du 2025; Omura 2018 Part 2 | Low Moderate methodological and adequacy concerns; the distinction between withholding and failed organisational follow-up is interpretive. |
