# Supplementary material for: Patterns and Potential Relationships of Organizational Silence Types Among Clinical Nurses: A Qualitative Meta‐Synthesis and Systematic Review
Source: Nurs Open. 2026 Nov 3;13(11):e71004. doi: 10.1002/nop2.71004 (PMC13731733; doi:10.1002/nop2.71004)
Supplement: Supplementary file 2 [file NOP2-13-e71004-s002.docx]

| **Item** | **Reporting topic** | **Manuscript location** | **Status and manuscript-based completion note** |
| --- | --- | --- | --- |
| 1 | Systematic review title | Title | Reported: the title explicitly identifies the report as a Systematic Review and Meta-Synthesis. |
| 2 | Abstract reporting | Abstract | Reported: objectives, eligibility, eight databases, search end date, synthesis and appraisal methods, 19 reports, findings, conclusions, funding and registration are present. Add a concise limitation of the included evidence; contextual concentration and incomplete reflexivity reporting are discussed only in the main text. |
| 3 | Review rationale | Background | Reported: prior qualitative synthesis and quantitative meta-analysis are discussed, with a rationale for examining motives, previous responses, communication choices and possible relationships. |
| 4 | Review objectives | Abstract, Objective; Background | Reported: the review aims to synthesise qualitative evidence on clinical nurses’ silence and examine potential relationships among motives, earlier responses and subsequent expression choices. |
| 5 | Eligibility and synthesis grouping | Methods, 1.5 Eligibility Criteria; 1.6 Data Extraction and Synthesis | Reported: population, phenomenon, design, language and report availability criteria are specified. Nurse-specific evidence is required from mixed samples. Findings are grouped through inductive thematic synthesis. |
| 6 | Information sources and search dates | Methods, Search Strategy | Reported: A comprehensive literature search was initially completed on 25 April 2026 in seven electronic databases: Scopus, Embase, PubMed, PsycINFO, CNKI, Wanfang Data, and VIP. In response to reviewer comments, the English-language search strategies were revised and expanded, and CINAHL was added as an additional information source. The revised searches were completed on 6 October 2026, with the final search covering all eight databases from inception to that date. |
| 7 | Search strategies and limits | Methods, Search Strategy; Table 1 | Reported: strategies for all eight databases are provided. The Chinese-database entries use generic Topic/keywords notation; confirm the actual database-specific fields and executed limits. Platform details and any language/date filters applied during searching should be explicit. Strategy execution was not verified in this checklist. |
| 8 | Selection process | Methods, 1.4 Study Selection and Supplementary Searching | Reported: the first author searched and screened titles/abstracts and full texts against prespecified criteria. This is a single-person screening process; independent duplicate screening must not be inferred. |
| 9 | Data collection process | Methods, 1.6 Data Extraction and Synthesis | Reported: two postgraduate nursing students from the same research group performed extraction and verification respectively; one extracted, the other checked original texts. Article sections and third-member consensus are specified. This is not independent duplicate extraction. No investigator-contact process is described. |
| 10a | Qualitative data sought | Methods, 1.5; 1.6; Results opening | Reported with qualitative adaptation: participant quotations and primary-study interpretations concerning withholding concerns and barriers to expression are sought; mixed-profession evidence must be attributable to clinical nurses. Numerical outcome measures and time-point effect estimates are not applicable. |
| 10b | Study and participant variables | Methods, 1.6; Table 2 | Reported: study characteristics are extracted; Table 2 identifies title, authors, country, language, year, design/analysis, aim, participants, sample size and primary themes. |
| 11 | Primary-study methodological appraisal | Methods, 1.7 Methodological Quality Appraisal; 1.6 | Reported with qualitative adaptation: the 10-item JBI qualitative checklist, item categories, independent appraisal by two people, third-member resolution, retention of all reports and absence of a score/cutoff are specified. JBI methodological appraisal is used rather than an intervention-trial risk-of-bias tool. |
| 12 | Numerical effect measures | Not applicable | Not applicable: this review synthesises qualitative accounts and interpretations; no risk ratios, mean differences or other numerical effect measures are estimated. |
| 13a | Eligibility for each synthesis | Methods, 1.5; 1.6; Results opening | Reported with qualitative adaptation: eligible reports and nurse-attributed findings are selected, coded and compared, and descriptive/analytical themes are developed inductively. There are no planned intervention-effect pooling groups. |
| 13b | Preparing data for synthesis | Methods, 1.6 | Reported with qualitative adaptation: extraction from specified sections, verification against originals, Chinese-language coding, checking translated quotations, separating evidence levels and retaining source/participant/context links are described. Missing summary statistics and numerical data conversion are not applicable. |
| 13c | Tabulating and displaying evidence | Methods, 1.4; 1.7; Tables 2 and 3; Results 2.1–2.4 | Reported: Tables 2 and 3 display characteristics and JBI item judgments, and results present source-linked quotations and themes. These displays are present, but their preparation and the organisation of study contributions to each finding |
| 13d | Synthesis approach and software | Methods, Study Design and Approach; 1.6 | Reported with qualitative adaptation: Thomas and Harden thematic synthesis integrates qualitative evidence while distinguishing evidence levels. NVivo 15.0, inductive line-by-line coding, constant comparison, descriptive themes and analytical themes are specified. Statistical pooling models and numerical heterogeneity estimators are not applicable. |
| 13e | Exploring variation across findings | Methods, 1.6; 4 Limitations | Reported with qualitative adaptation: cross-study comparison retains differences in motivation, context and behavioural expression and contradictory accounts. Limitations state that independent samples did not support detailed subgroup comparison. Statistical subgroup analysis, meta-regression and effect-size heterogeneity analysis are not applicable. |
| 13f | Sensitivity or robustness analyses | Not applicable | Not reported: source checking and retaining contradictory accounts are described, but no formal sensitivity re-synthesis is specified. Quantitative model sensitivity is not applicable. Qualitative sensitivity analysis is possible, so its absence must not be labelled inherently inapplicable solely because the review is qualitative. |
| 14 | Bias from missing findings | Methods, Search Strategy; 4 Limitations | Partial: language and grey-literature restrictions are reported, but no formal assessment of bias from missing qualitative findings is described. Funnel plots and Egger-type tests are not applicable; possible missing-findings bias remains relevant to a qualitative review. |
| 15 | Confidence assessment methods | 2Results | reported: CERQual ： Supplementary Table2 |
| 16a | Search and selection results | Methods, 1.4; Figure 1 | reported: the text gives 1270 records, 853 duplicates, 417 screened, 371 excluded, 46 full reports, 27 full-report exclusions and 19 included reports. Figure 1 contains conflicting numbers (including 844 duplicates, 426 records and 40 reports) and labels the final synthesis quantitative. Reconcile the diagram and text using the actual selection log. |
| 16b | Relevant full-report exclusions | Methods, 1.5; Figure 1 | reported: general exclusion criteria and an exclusion count are provided. No cited list of apparently eligible excluded reports with report-specific reasons is present. This remains applicable to qualitative systematic reviews. |
| 17 | Included report characteristics | Table 2; Methods, 1.4; Reference list | Reported: an embedded Table 2 contains 19 report entries, study characteristics and primary themes, and identifies the shared Omura participant sample and mixed-role populations. Clinical eligibility of the Jeong report is flagged in the table note and needs verification; that issue is separate from reporting the characteristics. |
| 18 | Primary-study appraisal results | Table 3; Methods, 1.7 | Reported with qualitative adaptation: the embedded JBI matrix gives all ten item judgments for each of 19 reports, with narrative acknowledgment of reflexivity and ethics reporting limitations. |
| 19 | Study-level numerical results | Not applicable to numerical estimates; qualitative evidence in Results 2.1–2.4 and Table 2 | Not applicable: no numerical outcomes, group summary statistics, effect estimates or confidence intervals are synthesised. As the qualitative counterpart, source-linked participant quotations and primary-study themes are presented in Results and Table 2. Qualitative evidence reporting is additionally addressed through ENTREQ. |
| 20a | Contributors to each finding | Results 2.1–2.4; Tables 2 and 3 | Reported：qualitative adaptation: citations/quotations identify contributors to the themes; report characteristics and appraisals are displayed separately. The manuscript does not explicitly summarise contributing-study characteristics and methodological limitations for each individual synthesis finding. |
| 20b | Statistical synthesis results | Not applicable | Not applicable |
| 20c | Results of variation investigations | Results 2.1–2.4; 4 Limitations | Reported with qualitative adaptation: contextual contrasts, differences in motivation and expression, and opposing accounts are described. Statistical subgroup/meta-regression and heterogeneity-test results are not applicable. The limitations explicitly acknowledge insufficient independent samples for detailed subgroup comparison. |
| 20d | Sensitivity analysis results | Not applicable | Not applicable |
| 21 | Results of missing-findings bias assessment | 4 Limitations; related restriction in Search Strategy | Reported : possible limitations of restricted languages and omission of grey literature are discussed. No finding-specific assessment of missing-findings bias is presented. Numerical publication-bias test results are not applicable. |
| 22 | Confidence in synthesis findings | 2Results | reported: CERQual ： Supplementary Table2 |
| 23a | Interpretation in relation to evidence | 3 Discussion, including 3.1–3.4 | Reported: the synthesis is interpreted against prior literature and social information processing theory, with distinctions among motives, responses, timing/routes and organisational constraints. Proposed relationships are framed as interpretive rather than established transitions. |
| 23b | Limitations of the included evidence | 4 Limitations; Methods, 1.7; Table 2 notes | Reported: concentration of 15 of 19 reports in Asian settings, limited independent subgroup samples, variations in reflexivity/ethics reporting and transferability are discussed. The outdated 11-study count from the previous version has been corrected. The wording about longitudinal observation should recognise Abrams’ longitudinal design while distinguishing it from observed silence-type transitions. |
| 23c | Limitations of review processes | 4 Limitations; Methods, 1.4 and Search Strategy | Reported: language and grey-literature restrictions are discussed. Single-person screening is described in the methods but its potential implications are not discussed; absence of a formal sensitivity analysis and of manuscript-reported confidence assessment is not addressed. |
| 23d | Practice and research implications | 3 Discussion; 4 Limitations; 5 Implications for Nursing Management | Reported: management approaches account for motives, earlier responses, urgency and communication routes; future longitudinal/mixed-methods research is proposed and alternative expression is distinguished from withholding. |
| 24a | Registration details | Methods, Protocol Registration; Abstract, Registration | Reported: PROSPERO CRD420261379111 and claimed prospective registration are stated. The public record, date and match to this review have not been independently verified in this checklist. |
| 24b | Protocol access | Methods, Protocol Registration | Reported：the PROSPERO platform is named as the access location. Add a direct record link and clarify whether it provides the full protocol or only the registration record; no separate protocol location is specified. |
| 24c | Protocol amendments | 2Results | reported: CERQual ： Supplementary Table2 |
| 25 | Review support and funder role | Funding; Abstract, Funding | Reported for financial support: the authors state that no specific grant/funding was received. A specific grant-funder role is not applicable where no grant funder exists. Nonfinancial support is not explicitly described; clarify if relevant. |
| 26 | Competing interests | Conflict of Interest | Reported: the authors declare no conflicts of interest. |
| 27 | Availability of review materials | No explicit location | Not reported: no availability statement identifies access to extraction forms/data, coding materials, synthesis records or other review materials. Statistical analysis code is not applicable, but availability of qualitative review materials remains applicable. |
